# Antiseptic Treatment of Croup

**Published:** 1886-11

**Authors:** 


### *ANTISEPTIC TREATMENT OF CROUP.*

The following is a plan of treatment recommended by M. Renon (*Journal de Med. de Paris*): The patient is placed in a well-ventilated room of medium size, the temperature of which is maintained at from 68° to 75° F. Upon an oil-stove is kept a vessel, of a capacity of two quarts, in which the water is constantly boiling. Into this put, every three hours, a tablespoonful of a mixture of salicylic acid, 56 parts; benzoic acid, 112 parts; carbolic acid, 280 parts, and alcohol, 468 parts. The stove is placed near the bed, and the steam impregnated with this mixture is conducted, by means of suitably arranged curtains, to the patient. The patient is kept in this atmosphere until the symptoms have entirely disappeared, and for two or three days after; and, if tracheotomy has been performed, until the wound is closed. A close watch should be maintained over the case, and if any symptoms of poisoning are manifested, the quantity of carbolic acid should be diminished.

